# Experimental studies on preparation of the porous and small-diameter poly(ε-caprolactone) external vascular scaffold and its degradability and biocompatibility

**DOI:** 10.1051/rmr/180001

**Published:** 2018-06-01

**Authors:** Qingyun Chen, Xia Jiang, Li Feng

**Affiliations:** 1 Key Laboratory of Transplant Engineering and Immunology, Ministry of Health, West China Hospital, Sichuan University, Chengdu 610041 PR China; 2 Regenerative Medicine Research Center, West China Hospital, Sichuan University, Chengdu, Sichuan 610041 PR China

**Keywords:** External vascular scaffold, poly(ε-caprolactone), degradability, biocompatibility

## Abstract

*Aim*: This study was aim to prepare a porous poly(ε-caprolactone) (PCL) biodegradable external vascular scaffold by dipping and leaching method, and to assess its mechanical property, degradability and biocompatibility. *Methods*: We used the PCL-1, PCL-2 as the raw materials and NaCl particles as the pore-forming agents to construct a porous PCL external vascular scaffold. We tested the mechanical property of the porous PCL external vascular scaffold. The degradability of the scaffold was studied in the presence of thermomyces lanuginosus lipase (TL lipase). After 1, 3, and 5, 7 days, the samples were taken out, and the pH of the media was measured. The form-stability of the scaffold was investigated by macroscopic observation and the microstructure of it was observed by SEM. The cytotoxicity of the scaffold was evaluated by CCK-8 assay. *Results*: PCL-1 could make a white integrated external vascular scaffold with uniform texture. When the concentration of NaCl was less than or equal to 50%, the tensile strength of the porous PCL-1 external vascular scaffolds were higher than 4.2 Mpa, which meet the demand of clinical vascular transplantation. With the degradation of the scaffold in the lipase media, the form-stability of the scaffold was seriously destroyed, the surface of the scaffold began to degrade with some honeycomb holes, and the pH of the media values were lower than the initial reading of 7.4. Rat adipose-derived stem cells (rADSCs) cultured in the extractions of the porous PCL external vascular scaffold had good proliferation and cell morphology compared to the control group. *Conclusion*: The porous PCL-1-50 external vascular scaffold, with the 50% concentration of NaCl, had the maximum porosity on the basis of enough mechanical strength which meets the demand of clinical vascular transplantation. Moreover, it had good biocompatibility with rADSCs and the degradation mechanism of the scaffold was surface degradation.

## 1 Introduction

Vascular grafting is needed for those patients with severe cardiovascular disease such as coronary heart disease. The clinical application of autologous blood vessel transplantation, as the “gold standard” for vascular transplantation, is limited because of the inconvenience of harvesting and insufficient availability in patients with atherosclerotic disease [1,2]. At present, the artificial vascular scaffold, as an alternative of autologous blood vessel, had been widely used in clinical vascular surgery.

Usually, artificial vascular scaffold are mainly made of polymer materials. And most of them are nondegradable with the diameter greater than six millimeters, while the scaffolds less than six millimeters are challenged by long-term patency after transplantation [3,4]. Accordingly, there has been a great deal of researches toward constructing a tissue-engineered vascular graft which composed by seed cells and degradable polymer scaffolds. Before the scaffold degradation, it allows seed cells to grow around them with the stimulation of various growth factors and induces neovascularization. After a few months, the injured blood vessels will be repaired biologically. However, the research on tissue-engineered vascular graft still stay in the laboratory study stage, and there is currently no biodegradable external vascular scaffold product available for clinic use. Therefore, the main focus of tissue engineering vascular research is to prepare a biodegradable external vascular scaffold that meets the clinical demand with good mechanical property, degradability and biocompatibility.

Poly(ε-caprolactone) (PCL) has been approved by the Food and Drug Administration (FDA) of the United States for its applications in the field of Medicine with its good mechanical property, biodegradability and biocompatibility [5–7]. Based on it, PCL has the potential to prepare a biodegradable external vascular scaffold for clinical vascular grafts. The biodegradability of biomaterials is a key feature of its use in tissue engineering scaffolds. However, PCL degrades very slow and has a total degradation of 2–4 years depending on its initial molecular weight [8]. The accelerating degradation of enzymes is one of the common methods to evaluate the degradation behaviors of polymer materials [9].

Accordingly, the main purpose of this study was to use PCL as the raw material to prepare a PCL biodegradable external vascular scaffold, and to assess its mechanical property, degradability and biocompatibility.

## 2 Methods

### 2.1 Materials

The Poly (ε-caprolactone) (PCL-1, Mn = 300 000.0; PCL-2, Mn = 80 000.0) was purchased from Jinan Daigang Biomaterial Corporation (China). Thermomyces lanuginosus lipase (TL lipase, >100 000.0 units/g) was purchased from Sigma company (USA). All solvents and salts were purchased from Chengdu Kelong Chemical Reagent Corporation (China). CCK-8 kit was purchased from Shanghai 7 sea biotech (China). Rat adipose derived stem cells were provided by the Regenerative Medicine Research Center (China).

### 2.2 Preparation of the porous PCL external vascular scaffold

In this study, the PCL external vascular scaffolds were prepared by dipping and leaching method [10]. Firstly, the PCL was dissolved in chloroform with a ratio of 1:10, and then sodium chloride salt particles (< 500 mesh) were added into PCL solution with 10 wt%, 30 wt%, 50 wt%, 70 wt%, 90 wt% of PCL. The cylindrical mold with a diameter of 5.0 mm was immersed in NaCl-PCL mixed suspension, and then it was dried after being taken out. After repeated dipping 10 times, we put it into the ultra-pure water to dissolve NaCl particles, and the water was changed every day. Until the chloride ion conductance values reduced to the same with the ultra-pure water and kept constant, the mould was taken down and the scaffolds were freeze dried.

### 2.3 Enzymatic degradation

The PCL external vascular scaffold was cut into strips with a length of 60.0 mm and a width of 5.0 mm, and was placed into a conical flask containing 25.0 mL of the TL lipase (1 mg mL^−1^) solution that was refreshed on alternate days. The degradation experiments were performed in triplicate at 37 °C. After 1, 3, and 5, 7 days, the specimens were taken out from the lipase media and washed with distilled water and then vacuum-dried at −90 °C to a constant weight.

### 2.4 Characterization

The pH of the degradation products was monitored by inserting a combination microelectrode (InLabMicro™, Toledo-Mettler) into the medium and following changes there with a digital pH meter (FiveEasy™, Toledo-Mettler). The form-stability of porous PCL external vascular scaffold was investigated by macroscopic observation and the microstructure change of which was observed by SEM with a FEI inspect F50 microscope (FEI, UAS). The mechanical properties of the PCL external vascular scaffolds were measured using an Instron 1121 universal testing machine (Instron, USA) with a crosshead speed of 50 mm/min. The tests were done on triplicate samples, and the results were presented as an average.

### 2.5 Cytotoxicity test

The cytotoxicity test was performed by an indirect contact method. Rat adipose-derived stem cells (rADSCs) were used to evaluate the cytotoxicity of the PCL external vascular scaffold. At first, rADSCs were cultured in the Dulbecco's modified Eagle's medium (DMEM) with 10% fetal bovine serum (FBS), 100 U mL^−1^ penicillin and 100 µg mL^−1^ streptomycin at 37 °C in a humidified atmosphere of 5% CO_2_. According to ISO 10993-12 [11], extraction medium was prepared using DMEM with a surface area/extraction medium ratio of 1.25 cm^2^ ml^−1^ in a humidified atmosphere with 5% CO_2_ at 37 °C for 24 h. After the extracts were centrifuged, the supernatant fluid was withdrawn and stored at 4 °C before cytotoxicity test.

The cells were incubated in the 96-well plates at the density of approximately 1 × 10^3^ cells per 100 µl medium in each well and incubated for 24 h to allow attachment. DMEM was then sucked out and replaced by extraction medium. After 2, 3, and 5, 7 days' incubation in the incubator respectively, the 96-well plates were observed under an optical microscope. Thereafter, 10 µl of CCK-8 was added to each well. The cells were incubated with CCK-8 for 2 h. The absorbance of each well was tested by a microplate reader (Bio-RAD680) at 450 nm.

### 2.6 Statistical analysis

Data were analyzed using SPSS 19.0 and were expressed as mean ± standard deviation. The differences between the groups were compared using the *t* test; *P* value  < 0.05 was statistically significant.

## 3 Results

### 3.1 Preparation and appearance of the PCL external vascular scaffold

**Fig. 1:**
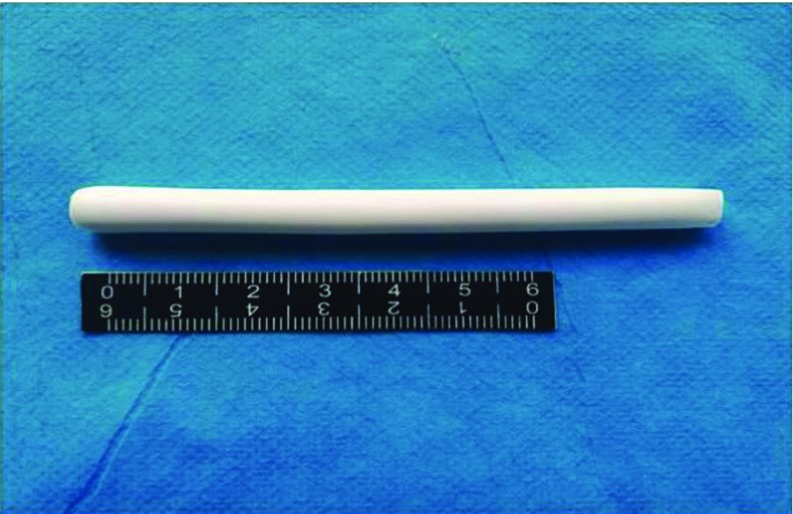
The macroscopic observation of the porous PCL-1 external vascular scaffold.

The PCL external vascular scaffolds were prepared by dipping and leaching method. We choose PCL-1 and PCL-2 as the raw materials (Tab. 1). Our results found that PCL-2 (Mv = 80 000.0) compare to PCL-1 (Mv = 350 000.0) couldn't fabricate an external vascular scaffold because the strength was too low to be formed. PCL-1 could make a white integrated external vascular scaffold with uniform texture (Fig. 1), which inner diameter was 5.0 mm and thickness was 0.5–0.6 mm.

**Table 1:** The intrinsic viscosity and molecular weight of PCL.

Samples	Intrinsic viscosity(dl/g)	Molecular weight
PCL-1	2.0–3.0	300 000.0
PCL-2	0.5–1.0	80 000.0

### 3.2 Mechanical properties of the porous PCL-1 external vascular scaffolds

**Fig. 2:**
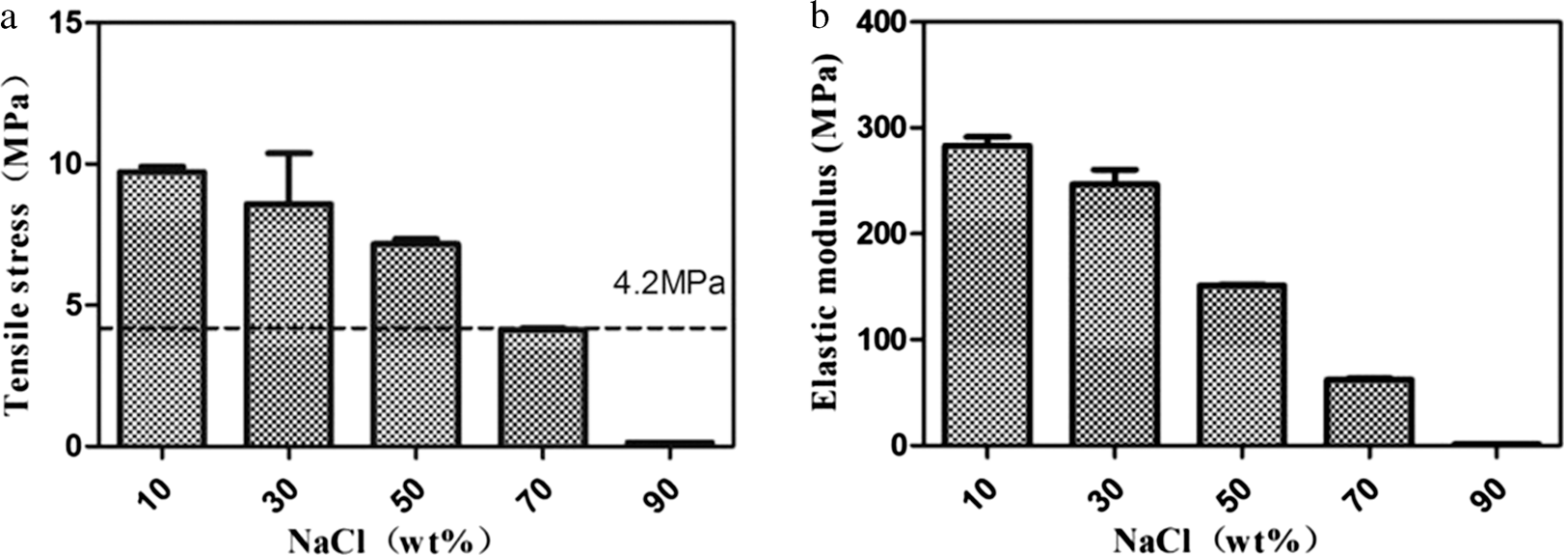
The tensile stress and elastic modulus of the porous PCL-1 external vascular scaffold prepared with different concentration of NaCl. Data are mean ± standard error for *n* = 3 replicates.

It has been reported in the literature that the tensile strength of external vascular scaffolds that can be used for vascular grafting should be higher than 4.2 MPa [12]. Our results showed that with the increase of NaCl concentration, the tensile strength and elastic modulus of the PCL-1 external vascular scaffold decreased gradually (Fig. 2). When the concentration of NaCl was less than or equal to 50%, the tensile strength of the porous PCL-1 external vascular scaffolds were higher than 4.2 Mpa (Fig. 2a), and its mechanical strength met the requirements of vascular grafts on clinic. All of them, the PCL-1-50 external vascular scaffold, with the 50% concentration of NaCl, had the maximum porosity on the basis of enough mechanical strength.

### 3.3 Degradation characteristics of the porous PCL-1-50 external vascular scaffold

#### 3.3.1 Form-stability

**Fig. 3:**
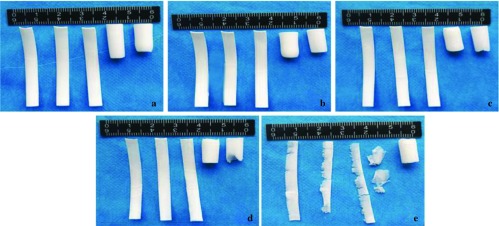
The macroscopic observation of the PCL-1-50 external vascular scaffold in different degradation times, a: 0d, b: 1d, c: 3d, d: 5d, e: 7d.

Figure 3 showed the macroscopic observation of the PCL-1-50 external vascular scaffold after incubating in TL lipase in different time. As the degradation progressed, the scaffold structure was gradually destroyed. After 7 days of degradation, the structure of the scaffold was severely damaged (Fig. 3e).

#### 3.3.2 Surface morphology

**Fig. 4:**
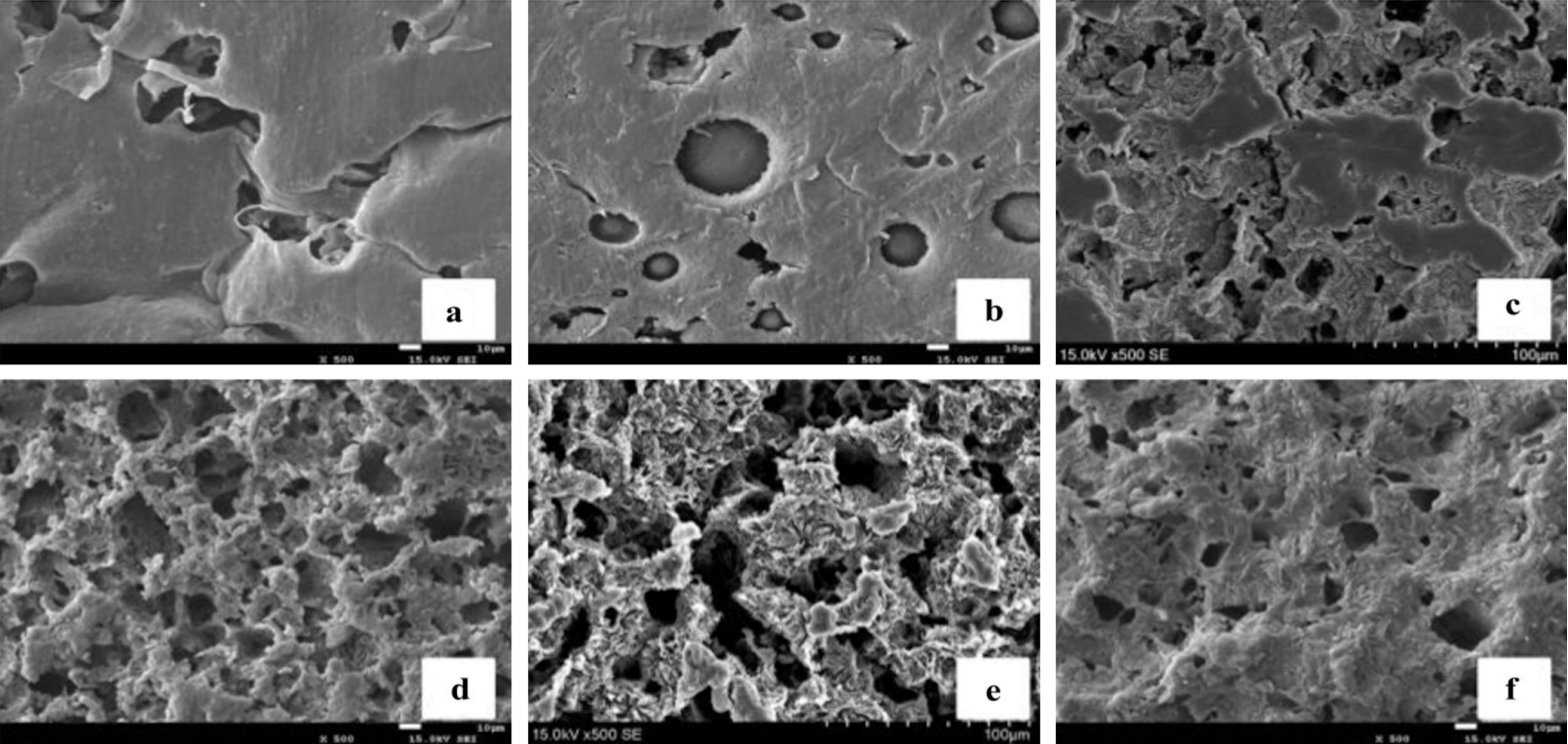
The microstructures of the porous PCL-1-50 external vascular scaffold in different degradation times (×500), a: 0d, b: 10 min, c: 1d, d: 3d, e: 5d, f: 7d.

The microstructures of the PCL-1-50 external vascular scaffold before and after enzymatic degradation were shown in Figure 4. The surface of the PCL-1-50 external vascular scaffold initially appeared rather smooth before enzymatic degradation (Fig. 4a). After 10 min, small honeycomb holes began to appear on the surface (Fig. 4b). As the degradation progressed, the erosion of the scaffold became remarkable (Fig. 4d–f). The results indicated that the degradation mechanism of the PCL-1-50 external vascular scaffold mainly was surface degradation.

#### 3.3.3 Changes in pH of the lipase solution

**Fig. 5:**
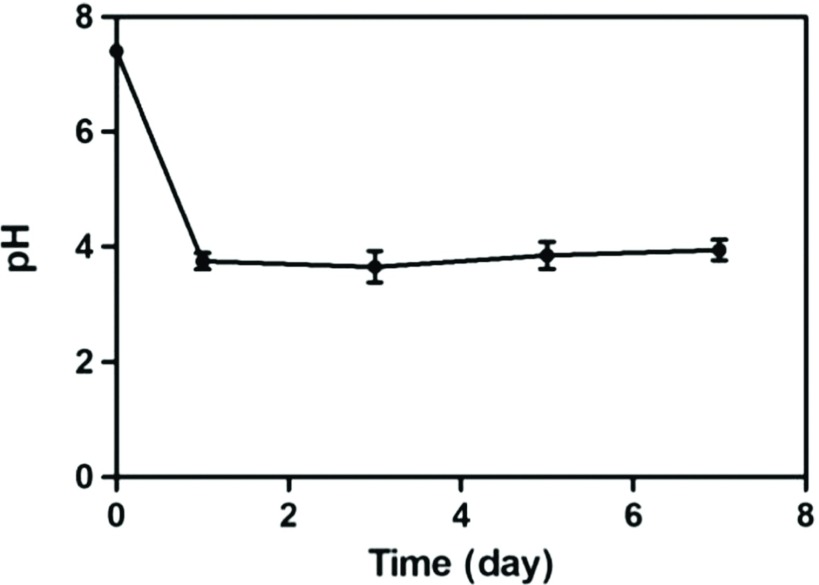
The changes of pH in media in different degradation time. Data are mean ± standard error for *n* = 3 replicates.

Figure 5 showed the change of pH values in media as the scaffolds degradation occurred. It was obvious that the pH values were all lower than the initial reading of 7.4 at every time point and stabled at around 4.0.

#### 3.3.4 Mechanical properties

**Fig. 6:**
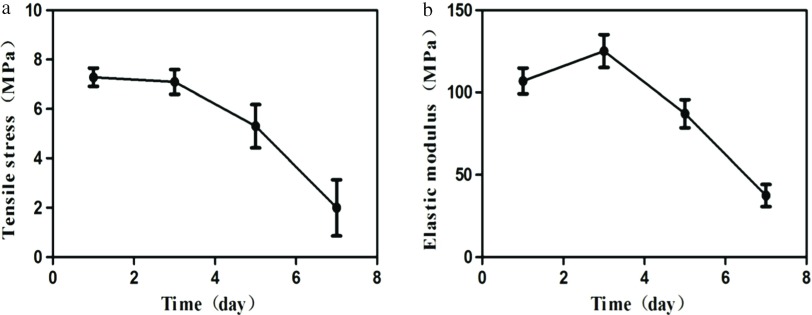
The tensile stress and elastic modulus of the porous PCL-1-50 external vascular scaffold in different degradation times. Data are mean ± standard error for *n* = 3 replicates.

Mechanical properties tests results showed that the tensile strength of the porous PCL-1-50 external vascular scaffold decreased gradually with the time of degradation (Fig. 6a). After 7 days, the macrostructure was severely damaged and the tensile strength was less than 4.20 MPa, which was not sufficient to meet the mechanical properties required for vascular grafts. In addition, in the process of degradation, the elastic modulus showed a tendency to rise first and then decrease (Fig. 6b).

#### 3.4 Cytotoxicity evaluation

**Fig. 7:**
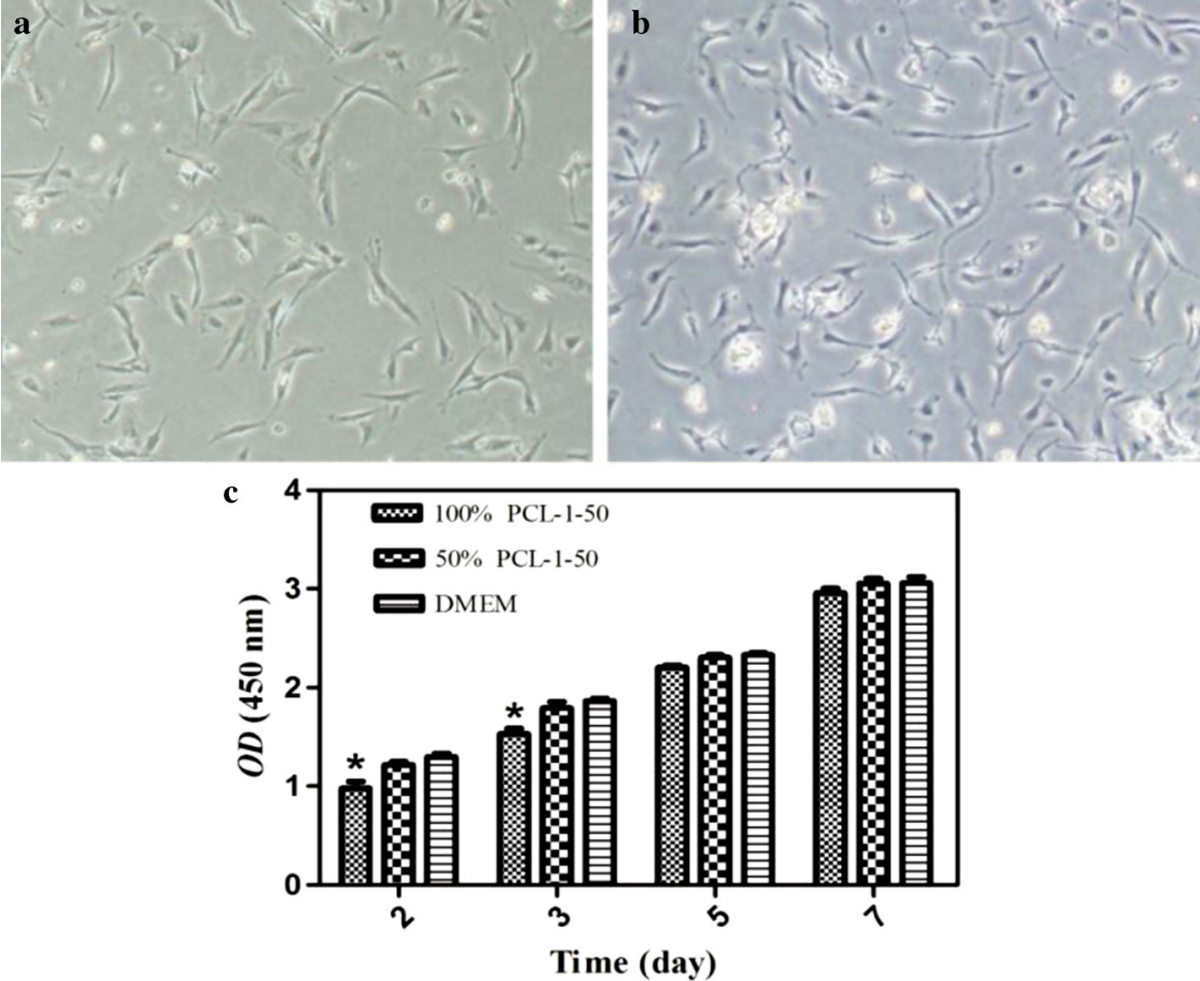
The cell morphology and proliferation of rADSCs cultured in the porous PCL-1-50 external vascular scaffold extractions. The morphology of rADSCs cultured in (a) the 100% scaffold extractions and (b) the DMEM for 3 days; (c) Proliferation of rADSCs cultured in the 100%, 50% scaffold extractions and DMEM for 2, 3, 5, and 7 days. Data are mean ± standard error for *n* = 6 replicates, ^*^p < 0.05.

Cytotoxicity was performed at days 2, 3, 5 and 7 of culture to evaluate the cytotoxicities and biocompatibilities. The results indicated that the rADSCs showed good proliferative activities to the extractions of the porous PCL-1-50 external vascular scaffolds, and were in a logarithmic growth phase for 7 days' culture (Fig. 7c). The morphology of rADSCs remained normal after being cultured in the 100% scaffold extractions for 3 days compared with the control group, showing long fusiform shape, strong refraction and good adherent (Fig. 7a and b).

## 4 Discussion

Since the long-term patency rate of non-biodegradable vascular scaffold with small caliber in vascular transplantation is not high, a great deal of researches has begun to construct a tissue-engineered blood vessel composed with biodegradable synthetic materials [13].

In this experiment, we used PCL as the raw material and NaCl particles as the inside pore-forming agent. We could adjust the porous structures and mechanical properties of the scaffold according to the concentration of pore-forming agent. The porous structure of the external vascular scaffold is conductive to cell attachment, growth and angiogenesis [14]. The PCL-1-50 external vascular scaffold, with the 50% concentration of NaCl, had the maximum porosity on the basis of enough mechanical strength which could meet the demand of the clinical vascular transplantation.

The biodegradability of PCL is a key characteristic for its application in the field of tissue engineering scaffolds, and a lot of studies had reported the good performance of lipase in degrading PCL, including thermomyces lanuginosus lipase, pseudomonas lipase and porcine pancreatic lipase [15–17]. In our experiment, we studied the degradation performance of the porous PCL-1-50 external vascular scaffold in the presence of TL lipase.

The degradation mechanism of polymer materials can be divided into surface degradation and bulk degradation [9]. The surface degradation means the degradation occurs on the surface of the material firstly. This typically results in thinning of the polymer and reducing slowly of the molecular weight. Nevertheless, bulk degradation occurs in the inner polymer is characterized by little change of the volume and decreasing significantly of the molecular weight. In this research, After 7 days degradation, the viscosity average molecular weight of the PCL-1-50 external vascular scaffold reduced slowly (M_η0_ = 300 000.0, M_η7_ = 217 141.7). Moreover, the SEM results showed that the surface of the scaffold began to degrade firstly. Therefore, the degradation mechanism of the PCL-1-50 external vascular scaffold mainly was surface degradation.

In addition, the relevant researches had suggested that the pH value of the degradation buffer solution would be reduced by the fracture of the ester bond and the release of a large number of carboxylic groups during the degradation process of PCL [18]. Our results of this experiment also confirmed this view. In the process of degradation, the degradation buffer was changed every other day. Acid buffer solution containing degradation products was taken out to measure the pH value each time, and the same volume of fresh degradation buffer was added to and incubated at the same time. Based on it, the pH value of the buffer solution didn't continue to decline, and had been stable at around 4.0 during the process of degradation. Furthermore, the pH value at each time point was close, indicating that the amount of released acid products was the same at the each time point. According to the degradation kinetics [19], the PCL-1-50 scaffold was conformed to the zero order kinetics with respect to the TL lipase concentration.

After the biodegradable scaffold implanted in vivo, the mechanical properties will change with the degradation of the scaffold. Before the completion of vascular repair, the scaffold must maintain a certain mechanical properties to support vascular tissue regeneration and maintain blood flow patency. Therefore, it's important to observe the mechanical properties' change of the scaffold during the degradation. Our results showed that the tensile strength of porous PCL-1-50 external vascular scaffold decreased gradually during the enzymatic degradation. After 7 days of degradation, the tensile strength of the scaffold was less than 4.20 MPa, which was not sufficient to meet the mechanical properties required for vascular grafts on clinic. The elastic modulus showed a tendency to rise first and then decrease. Elastic modulus was an elastic deformation measure index for material. The larger the value was, the smaller the elastic deformation was, and that is, the material was greater stiffness [20]. The increase of elastic modulus during degradation indicated that the porous PCL-1-50 external vascular scaffold hardened during degradation. The value of the elastic modulus may be related to the characteristic of semi-crystalline of PCL. During the degradation of the porous PCL-1-50 external vascular scaffold, the non-crystalline areas degraded first, inducing increase of the crystallinity, which affected the elastic modulus of the scaffold.

Constructing of a tissue engineered blood vessels are built on the basis of good seed cells and well-performing biomaterials, and the biomaterials should have good biocompatibility with seed cells [21]. rADSCs are a type of cells with multipotential differentiation, which can differentiate into vascular cells such as smooth muscle cells and endothelial cells [22]. Compare to smooth muscle cells, endothelial cells and other stem cells, rADSC has the advantages of no immune rejection, no ethical controversy, wide range of sources, convenience to obtain and low donor damage [23]. It has the potential to be seed cells for tissue engineering blood vessels. In vitro cytotoxicity test is the main method for evaluating biocompatibility of biomaterials, which includes direct contact experiment and indirect contact experiment, and both can qualitatively or quantitatively evaluate the cytotoxicity of the material [24,25]. In this experiment, we choose the indirect contact experiment, i.e., scaffold extractions experiment. Our results showed that the porous PCL-1-50 external vascular scaffold had good biocompatibility with rADSCs.

Subsequently, our team will choose adipose stem cells of rhesus monkey as seed cells, and combine them with the porous PCL-1-50 external vascular scaffold to construct a tissue engineering blood vessel. And then, vascular grafting was performed on the abdominal aorta of the rhesus monkey and to verify the vascular repair role of the scaffold.

## 5 Conclusion

The porous PCL-1-50 external vascular scaffold, with the 50% concentration of NaCl, had the maximum porosity on the basis of enough mechanical strength which meets the demand of clinical vascular transplantation. Moreover, it had good biocompatibility with rADSCs and the degradation mechanism of the scaffold mainly was surface degradation. It has the potential to be used in clinical vascular grafts in the future.

## Conflicts of interest

All authors certify that they have no financial conflicts of interest.
